# QTL architecture of vine growth habit and gibberellin oxidase gene diversity in wild soybean (*Glycine soja*)

**DOI:** 10.1038/s41598-019-43887-z

**Published:** 2019-05-14

**Authors:** Ruikai Wang, Li Liu, Jiejie Kong, Zhiyong Xu, Javaid Akhter Bhat, Tuanjie Zhao

**Affiliations:** National Center for Soybean Improvement/Key Laboratory of Biology and Genetics and Breeding for Soybean/State Key Laboratory for Crop Genetics and Germplasm Enhancement, Nanjing Agricultural University, Nanjing, 210095 China

**Keywords:** Agricultural genetics, Agricultural genetics

## Abstract

Vine growth habit (VGH) is a beneficial phenotype in many wild plants, and is considered an important domesticated-related trait in soybean. However, its genetic basis remains largely unclear. Hence, in the present study we used an integrated strategy combining linkage mapping and population genome diversity analyses to reveal the genetics of VGH in soybean. In this regard, two recombinant inbred line (RIL) populations derived by crossing a common wild soybean genotype (PI342618B) with two cultivated lines *viz*., NN 86-4 and NN 493-1 were used to map quantitative trait loci (QTL) for VGH. Here, we identified seven and five QTLs at flowering stage (R1) and maturity stage (R8), respectively, and among them *qVGH-18-1*, *qVGH-18-2*, *qVGH-19-3*, *qVGH-19-4* were identified as major loci (*R*^2^ > 10% and detection time ≥2). However, *qVGH-18-2* was considered as a main QTL for VGH being consistently identified in both RIL populations as well as all growth stages and cropping years. Out of all the annotated genes within *qVGH-18-2*, *Glyma18g06870* was identified as the candidate gene and named as *VGH1*, which was a gibberellin oxidase (GAox) belongs to 2-oxoglutarate-dependent dioxygenase (2- ODD). Interestingly, there was one member of 2-ODD/GAox in *qVGH-18-1* and *qVGH-19-4* named as *VGH2* and *VGH3*, respectively. Moreover, from sequencing data analysis *VGH1* and three other *GAox* genes were found significantly divergent between vine and erect soybean with *F*_ST_ value larger than 0.25. Hence, GAox was assumed to play a major role in governing inheritance of VGH in soybean. Therefore, elucidating the genetic mechanism of GAox is very useful for exploring VGH and other stem traits, as well as genetic improvement of plant type in soybean.

## Introduction

Annual wild soybean (*Glycine soja* Sieb. & Zucc.) is an ancient species of cultivated soybean (*Glycine max* L.). It has many useful traits including high protein content, small seed size and resistance to biotic as well as abiotic stresses, which are very important for soybean crop improvement^1^. However, some traits such as vine stem, pod shattering, bloom and black seed coat in wild soybean are undesirable for soybean breeding program. Vine growth in wild soybean is characterized with the slender and twining stem on some supports during plant growth. Twining habit has been proposed to describe the performance of wild soybean stem^2^. In fact, the degree of vine stem varied from erect stem without twining in most cultivated soybean to completely twining in most wild soybean^3^. It is always negatively related to plant lodging degree in crop production. On the other hand, vine growth is an adaptation characteristic for climbing plants with some advantages of plant dispersal, water transport, growth and survivor in natural environments^4^. It is a complicated trait involving many morphological characteristics. In watermelon, the vines related traits included stem growth (upright or bending), leaf size, leaf margin (circular or pointed) and plant size^5^. Many wild relatives of legume including wild soybean and perennial wild soybean have vine stems. In common bean, vine growth habit (VGH) has three forms viz., Type I (determinate bush), Type II (indeterminate upright), and Type III (indeterminate vine)^6^, indicating the importance of both stem termination type and twining degree for VGH.

In soybean, vine stem is a quantitative and dominant trait always measured by grading index or winding characteristics^3^. In an inter-specific recombinant inbred population (RIL), two QTLs *viz*., *qTH-D1b* and *qTH-G* located on chromosomes 2 and 18, respectively were found to regulate the twining habit in mature period of soybean^3^. The nearest marker Satt235 of *qTH-G* was also identified as target marker of QTL for plant height, number of nodes and maximum internode length^3^. VGH is also affected by external environment in soybean. For example, soybean of less shade tolerant will grow slender and lodging stems in the shaded field environment^7^.

There are three stem growth habits reported in soybean *viz*., indeterminate, semi-determinate and determinate. The terminal bud of indeterminate stem type will continue the vegetative activity of shoot apical meristem (SAM) during most of the growing season^8^. For the opposite determinate stem, the vegetative activity of SAM ceases when it becomes an inflorescence. Two genes, *Dt1* and *Dt2*, control the stem termination types in soybean. The *Dt1* gene is ortholog of Arabidopsis *TERMINAL FLOWER 11* (*TFL11*)^9,10^. The *Dt1* allele might control the determinate habit *via* the earlier loss in *GmTFL1b* expression concomitant with floral induction, although it functions normally under the non-inductive phase of flowering. *Dt2* is a gain-of-function of MADS-domain factor gene that specifies semi-determinacy in soybean. This gene (*Dt2*) appears to repress the expression of *Dt1* in the SAMs to promote early conversion of the SAMs into reproductive inflorescences^11^.

As universally known gibberellins is a class of essential hormones controlling a variety of growth and developmental processes during the entire life cycle of plants. It is very important for plants to produce and maintain optimal levels of bioactive GAs to ensure normal growth and development. The gibberellin biosynthesis pathway involves the precursor of trans-geranylgeranyl diphosphate, and synthesizes GA4 at last in a series of enzyme-substrate reactions^12^. The bioactive GAs synthesized in higher plants includes GA1, GA3, GA4 and GA7, while most of the other GAs are not bioactive^13^. Transformation of different GAs depends on GA oxidation reaction. Gibberellin oxidases (GAoxs) belonging to the 2-oxoglutarate-dependent dioxygenases (2-ODDs) catalyzes the later steps in the biosynthetic pathway of particularly importance in regulating bioactive GA levels, which includes GA20ox, GA3ox and GA2ox^13^. Moreover, GA20ox catalyses the multi-step oxidation of GA12 and GA53 to form the C19 skeleton, while GA3ox consecutively produces the final bioactive products *viz*., GA4 and GA1^14^. However GA2ox is involved in inactivation of GA, and act against either bioactive GAs (GA4 & GA1) or their immediate C19 precursors (GA9 and GA20)^15^ or against C20-GAs earlier in the pathway (e.g., GA12 and GA53)^16^. In other words, GA20ox and GA3ox produce bioactive GAs while GA2ox transforms them into non-bioactive GAs.

The mutants of *GA*20*ox* and *GA3ox* genes are different from that of *GA*2*ox*, due to their different functions. As GA20ox and GA3ox produce bioactive GAs, functional deficient mutants of them show typical GA-deficient phenotypes, such as dwarfism and small dark green leaves^12,17,18^. However, functional deficient mutants of GA2ox exhibit constitutive activation of GA responses just opposite from GA20ox and GA3ox, showing longer and slender stem in rice^19^, barley^20^, Switchgrass^21^ and pea^22^.

By keeping the above into view, the present study has been used to conduct linkage mapping for dissecting genetic architecture of VGH using two inter-species recombinant inbred line (RIL) populations and their high-density genetic maps, and subsequently candidate genes of the major QTL were screened out through the analyses of available genome sequence information and sequencing data of the parents. Moreover, some of the genes coding for 2-ODD/GAox located in the QTL regions were used to compare the genetic differences between vine and erect soybean genotypes. Hence, the relationship of VGH, GAs and GAox in soybean is analyzed to reveal the genetics of VGH in *Glycine soja*.

## Results

### Stem vine morphology of NJRINP and NJRI4P populations

Phenotypically wild soybean (PI342618B) is far different from cultivated varieties *viz*., NN 86-4 and NN 493-1 in plant type, terminal inflorescence and podding habit. The male parent (PI342618B) was at about Level 3.5 for VGH phenotype in the flowering period (R1 stage), and Level 4 in maturity period (R8 stage). However, the NN 86-4 and NN 493-1 have upright stem at both R1 and R2 stages, and produces cluster inflorescence on the top with big apical leaves during flowering stage. At the end when the stem stops growing, cluster pods grows out. In comparison, the PI342618B produce flowers continuously and grew dispersed pods.

**Figure 1:**
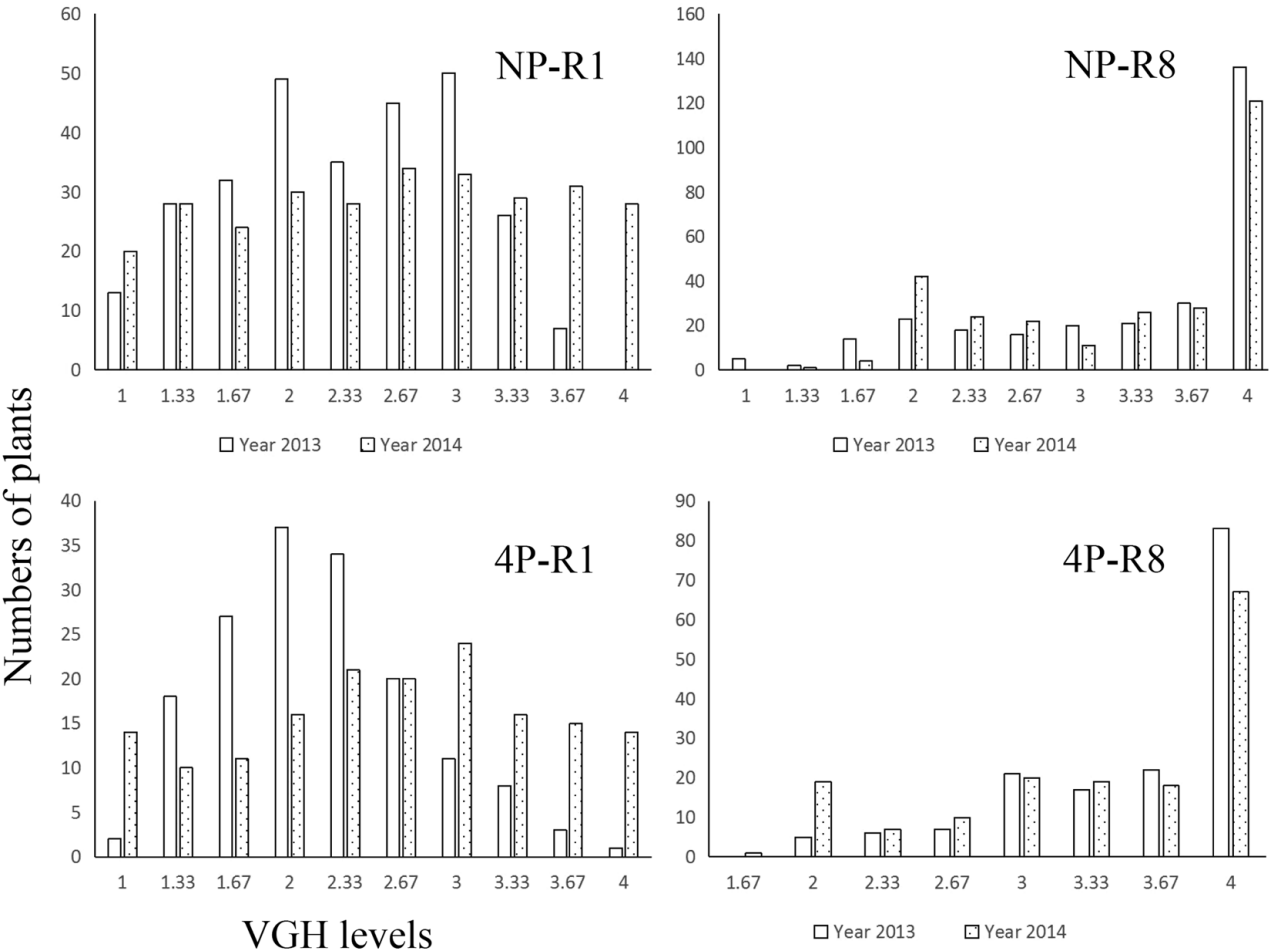
Frequency distribution of VGH in NJRINP and NJRI4P populations. R1 and R8, represent flowering and maturity stages of soybean growth period, respectively.

In NJRINP population, average VGH levels were 2.46 and 3.30 in R1 and R8 stages, respectively whereas it was 2.39 and 3.46 at R1 and R2 stages for NJRI4P population, respectively. Hence, our results revealed that there was more erect phenotype in flowering period (R1 stage) but more vine phenotype in maturity period (R8 stage) (Fig. 1). Furthermore, from the two year VGH phenotypic data, we observed that VGH phenotype had better reproducibility in R8 stage than in R1 stage. So it was recommended to map VGH genes at R8 stage.

### Identification of QTLs associated with VGH

By using the VGH phenotypic information and corresponding linkage maps constructed by 4354 molecular markers in NJRI4P population and 5728 in NJRINP population, we used composite interval mapping to identify QTLs for VGH in these two RIL populations. In R1 stage, seven QTLs were identified located on chromosome 1, 13, 18 and 19, whereas five QTLs were mapped on chromosome 18 and 19 at R8 stage (Table 1). However, the results obtained at R1 and R8 growth stages were considerably different, and some QTLs were specific to certain stage (Table 1). Out of them, four were defined as important (major) QTLs *viz*., *qVGH-18-1*, *qVGH-18-2*, *qVGH-19-3* and *qVGH-19-4* based on the value of *R*^2^ (≥10%) and detection time (≥2). The *qVGH-18-2* has been detected in both RIL populations, while the other three were detected only in NJRINP population. It was supposed that the genetic background of cultivated parent’s *viz*., NN 86-4 and NN 493-1 was different.

**Table 1:** QTLs detected in R1 (flowering) and R8 (maturity) stages of soybean for vine growth habit (VGH).

Growth stage	QTL	Pop-year	Chr. code	Position (cM)	LOD	Position	Additive effect	*R*^2^ (%)
*R1*	*qVGH-1-1*	NJRINP-2013	1	97.6	5.26	53225147–53345140	−0.16	5.23
	*qVGH-13-1*	NJRINP-2013	13	58.8	5.95	21992526–22153774	0.17	5.98
	*qVGH-13-2*	NJRINP-2013	13	68.1	6.87	27254556–27325385	0.19	6.85
	*qVGH-13-3*	NJRINP-2014	13	103.2	5.42	37848084–38017153	−0.21	4.86
	*qVGH-18-1*	NJRINP-2013	18	21.3	10.30	4007391–4143383	−0.24	11.56
		NJRINP-2014	18	20.9	10.42	3903511–4007390	−0.33	11.89
	*qVGH-18-2*	NJRINP-2013	18	29.3	13.61	5459178–5567920	−0.28	14.88
		NJRINP-2014	18	29.8	22.28	5506884–5614659	−0.46	23.25
		NJRI4P-2014	18	27.6	8.26	5786957–5924717	−0.34	14.03
	*qVGH-19-1*	NJRINP-2014	19	15.0	7.17	2003057–2106239	−0.24	6.48
*R8*	*qVGH-18-1*	NJRINP-2014	18	22.4	9.44	4214023–4364232	−0.3	10.07
	*qVGH-18-2*	NJRINP-2013	18	30.3	8.65	5567921–5686516	−0.24	7.10
		NJRINP-2014	18	30.9	14.16	5614660–5723037	−0.37	14.55
		NJRI4P-2013	18	26.8	6.13	5459148–5845684	−0.20	12.44
		NJRI4P-2014	18	27.6	8.88	5786957–5924717	−0.31	17.83
	*qVGH-19-2*	NJRINP-2013	19	22.9	7.57	3402304–3523254	−0.22	6.10
	*qVGH-19-3*	NJRINP-2013	19	89.6	32.24	44891230–45034862	−0.51	31.83
		NJRINP-2014	19	86.4	13.76	44117389–44287817	−0.37	13.93
	*qVGH-19-4*	NJRINP-2013	19	96.0	18.52	46858938–47231293	−0.40	20.29
		NJRINP-2014	19	96.0	6.61	46858938–47231293	−0.26	7.08

The *qVGH-18-2* was consistently identified in both RIL population and growth stages (R1 and R8) as well as in cropping years (2013 and 2014), except at R1 stage of 2013 in NJRI4P, because in this year the crop was subjected to unpredicted water-logging. Moreover, *qVGH-18-2* was located on chromosome 18 at about 27 cM in NJRI4P or 30 cM in NJRINP. It was reported to span a region of 227 kb from 5459177 to 5686516 bp by comprehensive analysis of mapping results in two RIL populations. In NJRINP, *qVGH-18-1* was identified at both R1 and R8 stages and were located in about 21 cM of chromosome 18. In addition, *qVGH-19-3* and *qVGH-19-4* were found in NJRINP population at R8 stage only, and were located at about 86–89 and 90 cM, respectively. These two QTLs were identified in both the studied years (2013 and 2014) (Table 1).

In summary, the QTLs of VGH were present on G and L linkage groups of soybean (chromosome 18 and 19). The *qVGH-18-2* was the main QTL for VGH in the both RIL populations, being persistently identified at both growth stages and years. Furthermore, *qVGH-18-1* and *qVGH-18-2* were concentrated at 20–30 cM of chromosome 18 (in RILs of NJRINP and NJRI4P), while *qVGH-19-3* and *qVGH-19-4* at 86–96 cM of chromosome 19 (in RILs of NJRINP) (Table 1).

### Candidate genes for *qVGH 18-2*

**Figure 2:**
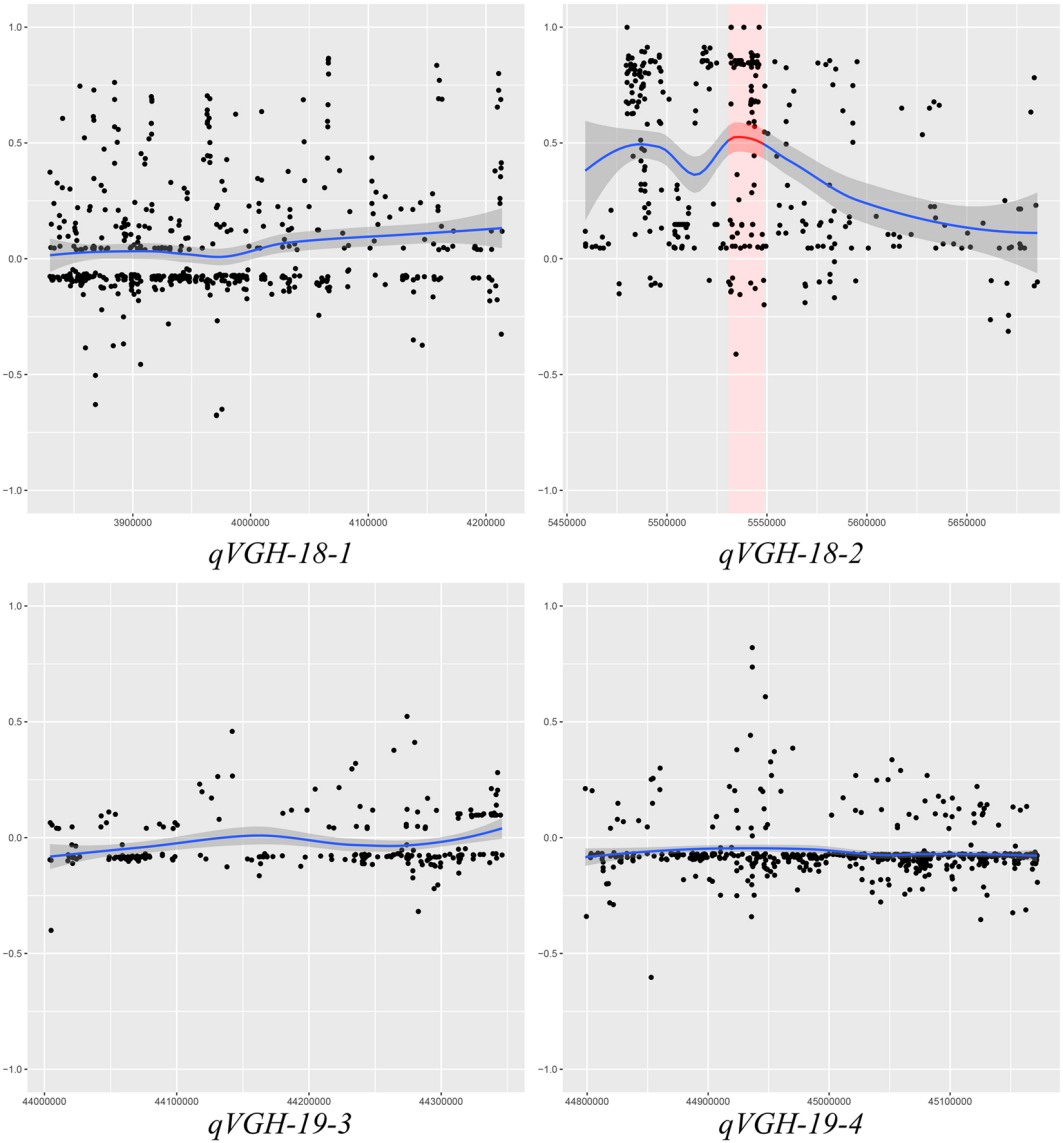
Sequence divergence between vine and erect soybean genotypes in the regions of *qVGH*. Note: Sequencing data of 18 vine growth and 14 erect soybean genotypes were used for divergent SNP mining in *F*_ST_ analysis at P value < 0.05. The data was published by Lam *et al*.^23^. Genome region of *VGH1* was indicated by red in *Glyma18g06870*: 5537666–5547946.

In the 227 kb region of *qVGH18-2*, 16 genes were annotated (Table S1). In order to further analyze these genes, a set of re-sequencing data for 18 vine and 14 erect soybean genotypes were used to find divergent gene^23^. In population genetics, *F*_ST_ provides a method to detect the evolutionary processes influencing the structure of genetic variation within and among populations^24^. By comparing SNPs between 18 vine and 14 erect soybean genotypes in the four QTLs, a high *F*_ST_ value was found in *qVGH-18-2* revealing considerably differentiation (Fig. 2). However, no such differentiation was reported for other three QTL regions *viz*., *qVGH-18-1*, *qVGH-19-3* and *qVGH-19-4* (Fig. 2).

**Figure 3:**
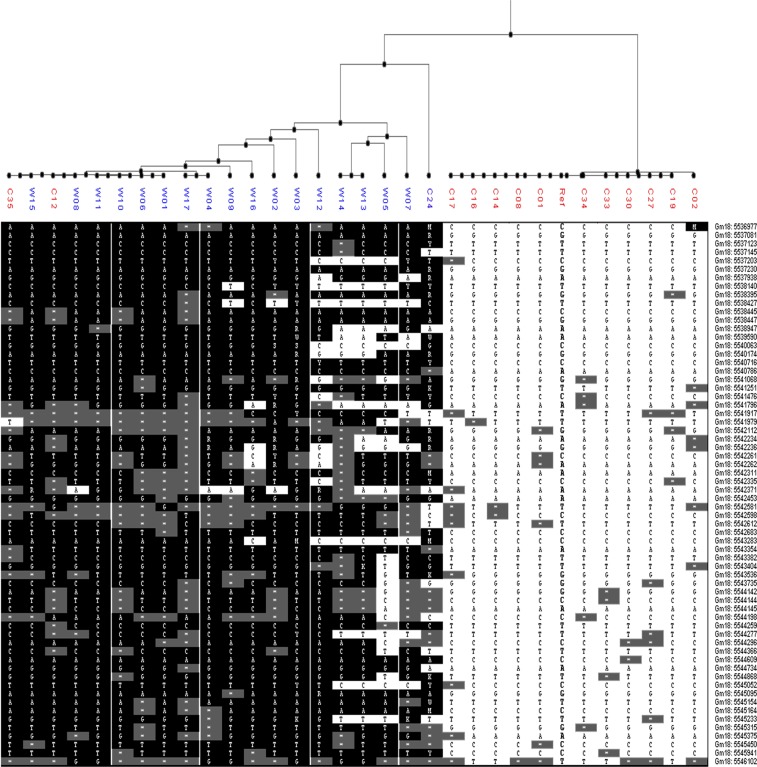
Sequence polymorphism at location of *VGH1* for 18 vine and 14 erect soybean genotypes. Note: 58 SNPs was used for polymorphism analysis. Vine soybean genotypes were in blue names, and the erect ones in red. The vine and erect soybean genotypes were cleanly divided into two groups except C12 and C35.

The SNPs of all the 16 genes in the region of *qVGH-18-2* are listed in Table S2. Among all the 16 annotated genes *Glyma18g06870* had a *F*_*ST*_ value of 0.67(Fig. 2; Table S2). Furthermore, there were total of 154 SNPs in the *Glyma18g06870*, out of which 58 SNPs was used for polymorphism analysis after low frequency and depth SNPs was filtered out (Fig. 3). Using this data the vine and erect soybean genotypes were clearly divided into two groups at *Glyma18g06870* (Fig. 3), and hence this was considered as a candidate gene of *qVGH18-2*, and was named as *VGH1*.

**Figure 4:**
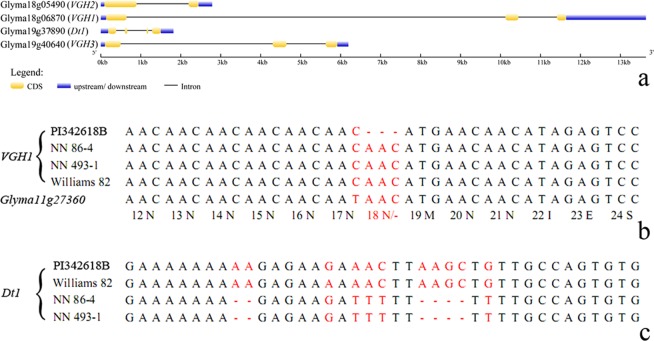
Gibberellin oxidases (*GAox*) genes in *qVGH* regions: (**a**) Gene structure of *GAox* and *Dt1* in *qVGH*; (**b**,**c**) Sequence polymorphism of *VGH1* and *Dt1* in Williams 82 and the three parents of RIL populations.

*VGH1* is a gibberellin oxidase (*GAox*) gene belongs to GA2ox subfamily of gibberellin oxidase. By gene sequencing, it was found that *VGH1* has a single codon deletion at 18N site in wild soybean (PI342618B), leading to an amino acid deletion in Exon 1 (Fig. 4b). However, the 18N site of *VGH1* has no such codon deletion for genotypes *viz*., NN 86-4, NN 493-1 and Williams 82. Furthermore, the gene *Glyma11g27360* is the closest homologue of *VGH1*. By sequence alignment, *Glyma11g27360* was found to have the same genotype as *VGH1* in case of NN 86-4, NN 493-1and Williams 82, indicating that the amino acid deletion in PI342618B emerged later (Fig. 4b).

By comprehensive analysis of mapping results, the other three *qVGH viz*., *qVGH18-1*, *qVGH19-3* and *qVGH19-4* were reported to span the chromosome regions of 460 kb, 143 kb and 372 kb, respectively (Table 1). It was noteworthy that one member of gene coding for gibberellin oxidase (GAox) were also found in *qVGH-18-1* and *qVGH-19-4*, which are *Glyma18g05490* and *Glyma19g40640*, respectively (Fig. 4a; Table S1). Furthermore, the *qVGH-19-3* possesses one gene as *Glyma19g37890* which is well known as soybean growth habit gene (*Dt1*)^9,10^. Both soybean growth and vine habit phenotypes are plant type characters. In the *Dt1* locus, the PI342618B and Williams 82 had the same haplotype genotype but different from NN 86-4 and NN 493-1. The difference is at Intron 1 which included six nucleotide residues insertion/deletion, and did not lead to any changes in amino acid residues (Fig. 4c). This type of mutation has been also earlier reported^9,10^.

### Sequence polymorphism of 2-ODD/GAox in wild and cultivated soybean genotypes

**Figure 5:**
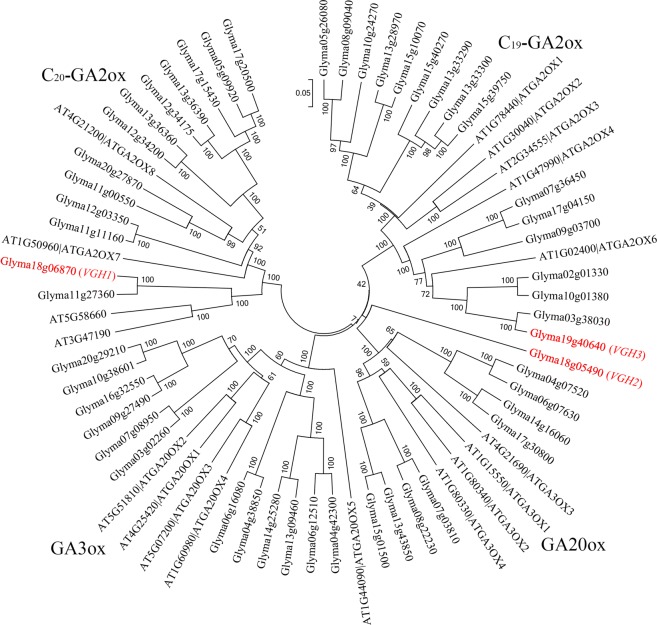
Phylogenetic relationship among the 2-ODDs of soybean and *Arabidopsis thaliana*, based on the amino acid sequence alignment. The phylogenetic tree is Neighbor-Joining (NJ) and bootstrap support is based on 1000 replicates. Note: The candidate genes *Glyma18g06870* (*VGH1*), Glyma18g05490 (*VGH2*) and Glyma19g40640 (*VGH3*) of our three identified QTL regions are highlighted by red outline.

As GAox are important candidate genes of VGH, many members of which regulate the gibberellin content in plants. Therefore genome-wide analysis of 50 2-ODD/GAox were carried out. This analysis leads to the identification of 16 C_19_-GA2ox, 13 C_20_-GA2ox, 12 GA3ox and 9 GA20ox in soybean (Fig. 5). There were two and one members of 2-ODD/GAox on the chromosomes 18 and 19, respectively. Furthermore, the phylogenetic tree revealed that *VGH1* (*Glyma18g06870*), *VGH2* (*Glyma18g05490*) and *VGH3* (*Glyma19g40640*) belongs to C_20_-GA2ox, GA20ox and C_19_-GA2ox, respectively (Fig. 5). This indicates that there is not a major subfamily to regulate VGH in soybean. In other words, different GAox are regulating the VGH rather than a smaller group of GAox.

The main candidate gene *VGH1* was in mutant form in PI342618B, but in wild form in NN 86-4, NN 493-1 and Williams 82 (Fig. 4b). From the sequencing data analysis of 18 vine and 14 erect soybean genotypes, *VGH1* was also observed to be highly divergent between these contrasting phenotypes (Fig. 3). Moreover, there was one member of GAox present in three of major *qVGHs* identified. Therefore we assumed that some of the 2-ODD may be divergent between vine and erect soybean genotypes. Furthermore, the wild soybean always has VGH while cultivated ones possess erect one, hence VGH is a domestication characters. Therefore, sequence diversity analysis was conducted to verify this hypothesis.

Sequencing data of 2-ODDs from 18 vine and 14 erect soybean genotypes was used in polymorphism analysis. The differential SNPs between vine and erect soybeans were explored by *F*_ST_ analysis (Table S2). Finally, we identified four2-ODD genes that were significantly divergent between vine and erect soybean with a *F*_*ST*_ value larger than 0.25 (Table S2). They were *Glyma18g06870* (*VHG1*, *C*_*20*_*-GA2ox*), *Glyma04g38850* (*GA20ox*), *Glyma03g38030* (C_19_-GA2ox) and *Glyma18g05490* (GA3ox). It should be noted that *VGH1* was found the most divergent gene between vine growth soybean and erect ones. The average *F*_ST_ of GA2oxs and GA20oxs were −0.07 and 0.04, respectively. Hence, GA2ox was more divergent between vine type and erect soybean genotypes compared to GA20ox.

## Discussion

The VGH is a typical quantitative trait, which is highly influenced by environment, trait investigation method, varieties and so on. In our study, we observed more upright phenotype in flowering period (R1 stage) but more vine phenotype in maturity period (R8 stage) during the both cropping years. Furthermore, the results of QTL mapping studies for VGH trait were different in both R1 and R8 stage, as well as in NJRINP and NJRI4P populations. This phenomenon was also observed in other studies, for example Liu *et al*.^3^, reported that QTL identification for twinning habit (number of times that a main stem winds around a support) in soybean was highly influenced by cropping years. Therefore, mapping of QTLs for VGH needs to consider the influence of these factors. Furthermore, in the present study we suggested that it is better to investigate VGH trait of soybean at R8 than in R1 stage, because there was a better reproducibility of VGH in R8 stage (Fig. 1).

In the present study we detected *qVGH-18-2* as a main QTL for VGH because it was consistently identified in both cropping years and RIL populations as well as crop stages (R1 and R8), with a coefficient of determination (*R*^*2*^) ranging from 12% to 23%. Therefore, we assumed *qVGH-18-2* an important as well as major QTL emerged in our experiments. The other three QTLs of VGH were detected in NJRINP population only for 2–3 times, such as *qVGH-18-1*, *qVGH-19-3* and *qVGH-19-4*. However, *qVGH-19-3* and *qVGH-19-4* were detected only in R8 stage.

Many types of plant trait phenotypes were reported to be highly related to plant height and GA level^25^. Here, we assumed that VGH in soybean is positively correlated with plant height and GA level. In the present study, we reported *Glyma18g06870* (*VGH1*) as a candidate gene of *qVGH-18-2*, due to several reasons. Firstly, *Glyma18g06870* is a GA2ox gene which can inactivate bio-active GAs, and whose loss of function could increase the content of bio-active GAs, and thereby plant height. There may be a correlation between plant height and VGH, because it was reported that in shaded field environment, soybean of less shade tolerant will grow slender and lodging stems^7^. Furthermore, VGH trait may be influenced by overgrowth and GA level. For example, defective mutant of GA2ox induced slender stem in pea which is closely related species of soybean^22^. Secondly, in our study we identified four major QTLs for no less than two times, and interestingly there existed one member of GAox encoding gene in three of them. The GAox encoding gene in *qVGH-18-2* was *VGH1* belonging to C_20_-GA2ox gene subfamily, whereas GAox encoding genes in *qVGH-18-1* and *qVGH-19-4* were *VGH2* (sub-family GA20ox) and *VGH3* (sub-family C_19_-GA2ox), respectively. Moreover, *VGH1* revealed considerable polymorphism between vine growth and erect parents of the two RIL population. Lastly, *VGH1* gene was divergent between vine and erect soybean in a resequenced population (Fig. 3). Hence, *Glyma18g06870* (*VGH1*) may play major role in controlling phenotypic variation of VGH in soybean. Previously, the locus for *VGH1* in soybean was also reported near the marker Satt235 of *qTH-G*, which is target QTL for plant height, number of nodes and maximum internode length^3^.

Many plant type traits were proved to be highly related to gibberellin metabolism^26^. Hence, GAs may be the internal cause of VGH as well as other plant height related phenotypes. For example, the most well known “Green revolution” genes was loss-of-function of a rice GA20ox gene leading to dwarfness of rice varieties^27,28^. In addition, wild soybean with VGH was longer than cultivated soybean. Therefore, we assumed the VGH in soybean was due the result of stem elongation.

In one of our major identified QTL (*qVGH-19-3*), there was a well-known gene called *Dt1* responsible for soybean stem growth habit which is a member of MADS-domain factor gene. As vine growth habit (VGH) and stem growth habit are similar traits in phenotypic characteristics^9^. Both vine growth and indeterminate stem soybean genotypes (one type in stem growth habit) had long and thin stem, which referred to stem elongation^9^.

Although, GA2ox, GA3ox, GA20ox belong to the 2-ODD/GAox, but they are different in their function. The GA20ox and GA3ox produce bioactive GAs while GA2ox inactivates them^13^. So functional deficiency of GA20ox and GA3ox leads to decreased level of gibberellin content, and hence dwarf stems in many plants^12,17,18,29,30^, whereas functional deficiency of GA2ox leads to higher level of gibberellin content and leads to slender stems in many plants^19–22^.

The VGH is a beneficial trait for wild soybean, because it is helpful for wild soybean to twine on some objects to grow^2^. It is understandably that loss-of-function of GA2ox in the wild soybean would generate higher GA content and plant height, because GA2ox inactivates bioactive GAs. Furthermore, *VGH1* was defective in wild parent (PI342618B), and but functional in our cultivated soybean parents (NN 86-4 and NN 493-1) (Fig. 4b). Hence, we assumed that the wild soybean may get benefit from *VGH1*’s loss-of-function to increase bioactive GA level, resulting in higher plant height and thereby VGH phenotype. However, the mutation in the form of loss of a single codon at 18 N site in *VGH1* gene in wild soybean parent (PI342618B) were not found in the other 17 wild and 14 cultivated soybean genotypes. Thus, in this case loss-of-function of *VGH1* gene may occur due to mutation at other sites or due to different mutant type of GA2ox genes. Hence, any type of functional deficiency in GA2ox will lead to higher GA content.

By comparing the sequence information of *VGH1* with its highly homologous gene *Glyma11g27360* (Fig. 4b), it was observed that wild soybean (PI342618B) has a mutant haplotype with loss of one amino acid residue at 18N site. While the cultivated soybean genotypes *viz*., NN 86-4, NN 493-1 and Williams 82 have wild type. This indicates that *VGH1* had lost its function in wild soybean PI342618B, and in theory loss-of-function of GAox encoding gene will cause plant to grow longer. Hence, VGH was screened out in the domestication of cultivated soybean. Furthermore, some 2-ODD/GAox were differential between wild (vine) and cultivated (erect) soybean genotypes (Table S2). Four of them were found to have *F*_ST_ value larger than 0.25, out of which the *F*_ST_ of *VGH1* was highest that even reach to 0.68. The other three that has *F*_ST_ value larger than 0.25 are *Glyma04g38850* (GA20ox), *Glyma03g38030* (C19-GA2ox) and *Glyma18g05490* (GA3ox). These 2-ODD/GAox genes were divergent between vine and erect soybeans, and were related to domestication process of wild and cultivated soybeans.

## Conclusion

The present study was the first detailed investigation of VGH in soybean, where we used an integrated strategy combining linkage mapping and population genome diversity analyses to reveal the genetics of VGH in soybean. We identified four major QTLs *viz*., *qVGH-18-1*, *qVGH-18-2*, *qVGH-19-3* and *qVGH-19-4*, among which *qVGH-18-2* was considered as a main QTL being consistently identified in both RIL populations as well as all growth stages and cropping years. Interestingly, annotation revealed that one member of *GAox* was found in three of our four QTLs. Moreover, phylogenetic analysis of 50 2-ODD/GAox genes revealed that there was not a major subfamily that regulates gibberellin (GA) content in soybean. In addition, sequencing data analysis revealed that *VGH1* and three other GAox genes were significantly divergent between vine and erect soybean with *F*_ST_ value larger than 0.25. Therefore, GAox or 2-ODD were considered to be the possible candidates for governing the inheritance of VGH in soybean. Hence, future studies are needed involving different “*omics*” based approaches to get detailed understanding of GAox role in VGH and, their practical utility in genetic improvement of plant type in soybean.

## Methods

### Plant materials and phenotypic evaluation

PI342618B is a wild soybean with VGH trait as wells high stress resistance. Nearly all the cultivated soybean genotypes are erect ones. NN 86-4 and NN493-1 are popular cultivated soybean genotypes in middle and lower reaches of the Yangtze River of china, Hence are selected for constructing the two different mapping population with the common wild parent (PI342618B).

Two recombinant inbred line (RIL) populations derived from the cross between a common wild soybean accession (PI342618B) with two cultivated varieties (used as female parents) were used to map QTL for vine growth habit (VGH) in soybean. The female parents of the two RIL populations, namely NJRINP and NJRI4P are NN 86-4 and NN 493-1, respectively. The NJRINP population has 286F_6:10_ lines, while as NJRI4P have 161F_6:9_ lines in 2013.

The RILs and parents were planted at Jiangpu agricultural experiment station of Nanjing Agricultural University for two consecutive years *viz*., June 20, 2013 and June 22, 2014, respectively. All plant materials were grown in hill plots with three replications by using a randomized complete block design in each year. The plot size is 1 m in length between two rows and 0.8 m in width between two hills. The VGH of all plant materials was evaluated by using a four-level indicator system at R1-R2 (flowering period) and R8 (maturity) stages, respectively^31^. The detailed phenotypic standard is as following. Level 1, is erect type, whose plant growth was strong, and over 4/5 of the main stem erected upward. Parents NN 493-1 and NN 86-4 has typical Level 1 phenotype. While, Level 2 is semi erect type in which 3/5 of lower stem was erect, and also stem was undulate but not wound. Level 3 is semi vine type, where more than 1/5 of the lower stem was erect, and slender in the upper and partly wrapped. Level 4 is vine type, which was winded on bamboo and intertwined; wild soybean parent (PI342618B) was typical Level 4.

### QTL mapping for VGH

The high-density genetic linkage maps for NJRINP and NJRI4P populations contain 5,728 and 4,354 bin markers that were obtained from 89,680 and 80,995 single nucleotide polymorphisms (SNPs), respectively. The total genetic distance covered were 2204.6 and 2136.7 cM for NJRINP and NJRI4P populations, with an average distance of 0.4 and 0.5 cM between neighboring bins, respectively^32^.

QTL mapping for VGH was performed using composite interval mapping (CIM) method in WinQTL Cartographer 2.5^23^. A stepwise forward regression procedure was selected with a 10-cM window at a walking speed of 1 cM. The LOD threshold was calculated using 1000 permutations.

### Candidate gene analysis

The predicted genes were queried and annotated through the SoyBase website (http://www.soybase.org/). The DNA polymorphism information was obtained from the re-sequencing data of 18 wild (vine) and 14 cultivated (erect) soybean genotypes^33^, and was analyzed by using SNPViz software^34^.

### Sequence collection and phylogenetic analyses of gibberellin oxidases

Genomic data for the gibberellin oxidase (*GAox*) proteins were searched on web of SoyBase. The information for the soybean *GAox* genes, including accession number, chromosomal location, open reading frame length as well as number of exons and introns, were retrieved from SoyBase public database. All the collected GAox candidate genes were used for phylogenetic analyses. The amino acid sequences were aligned using the CLUSTAL program of Molecular Evolutionary Genetics Analysis (MEGA) 7.0^35^. The *F*_ST_ was calculated by Arlequin 3.5^36^ and draw in R package.

## Supplementary information

qVGH 18-1 (Sheet 1); qVGH 18-2 (Sheet 2); qVGH 19-3 (Sheet 3); qVGH 19-4 (Sheet 4)

QTL region (Shee 1); All 2-ODD (Sheet 2)
